# The role of pirfenidone in the treatment of idiopathic pulmonary fibrosis

**DOI:** 10.1186/1465-9921-14-S1-S5

**Published:** 2013-04-16

**Authors:** Vincent Cottin

**Affiliations:** 1 Hospices Civils de Lyon, Hôpital Louis Pradel, Service de pneumologie – Centre de référence national des maladies pulmonaires rares, Université Claude Bernard Lyon 1, Lyon, France

**Keywords:** Idiopathic pulmonary fibrosis, pirfenidone, forced vital capacity, clinical trial

## Abstract

Idiopathic pulmonary fibrosis (IPF) is a progressive disease, with a median survival time of 2–5 years. The search for effective treatment has involved numerous clinical trials of investigational agents without significant success. However, in 2011, pirfenidone was the first drug to be approved for the treatment of IPF in Europe. Four key clinical trials supported the efficacy and tolerability of pirfenidone.

In the two recently published Phase III CAPACITY trials evaluating pirfenidone (studies 004 and 006), patients with mild-to-moderate IPF were treated with pirfenidone or placebo. Study 004 and pooled analysis of primary endpoint data from both studies showed that pirfenidone significantly reduced decline in percent-predicted forced vital capacity (FVC) compared with placebo (p<0.005). Evidence of beneficial effects of pirfenidone treatment was also observed with regard to several secondary endpoints. Pirfenidone was generally well tolerated, with the most common side effects being gastrointestinal and photosensitivity. Data from the RECAP extension phase of the CAPACITY studies, where patients were treated with pirfenidone for up to three years, further support the manageable tolerability profile of pirfenidone. The efficacy data, coupled with long-term safety data, provide further evidence of a clinically-meaningful treatment effect with pirfenidone in patients with IPF.

## Background

Idiopathic pulmonary fibrosis (IPF) is a debilitating disease, occurring predominantly in adults of around 60–75 years of age,[1] with an estimated prevalence of IPF of 1.6–1.7/10,000.[2-4] The disease course is progressive and ultimately fatal, with a median survival of 2–5 years [5,6] – worse than a number of malignancies [7]. Progressive deterioration of pulmonary function occurs, which increasingly limits the patient’s ability to perform normal physical activities.[8] The speed and extent of this deterioration is often unpredictable,[9] with patients who generally appear to have stable disease often suffering episodes of acute exacerbation.[10,11]

A decline in both relative and absolute changes in the forced vital capacity (FVC) has been shown consistently to predict mortality in patients with IPF.[12-14] A decline in FVC of 10% or more in a six-month period is associated with a nearly five-fold increase in the risk of mortality.[12-15] However, whilst serial FVC measurements are a validated marker of chronic disease progression, and frequently used as an endpoint in clinical trials,[16-18] it is not a proven surrogate for mortality. Nevertheless, agents that attenuate the decline in FVC are anticipated to play an important role in IPF management.

Pirfenidone has been shown to reduce the decline in FVC in patients with IPF. This is an orally available drug that exhibits anti-fibrotic and anti-inflammatory properties *in vitro* and *in vivo*.[19-23] There is evidence to show that pirfenidone diminishes fibroblast proliferation, secretion of the fibrosis-associated proteins and cytokines, biosynthesis and accumulation of extracellular matrix as well as accumulation of inflammatory cells and tumour necrosis factor-α synthesis.[19-23]

## Clinical trials of pirfenidone in patients with IPF

Following evaluation in Phase II and Phase III clinical trials in patients with IPF.[24-26], pirfenidone was approved by the European Commission in February 2011 Pirfenidone is indicated for the treatment of patients with mild-to-moderate IPF. Mild-to-moderate disease was characterised in two pivotal Phase III studies using the following functional criteria: FVC ≥50% of predicted value, carbon monoxide diffusing capacity (DL_CO_) ≥35% of predicted value and a 6 minute walk test (6MWT) distance of ≥150 m.[26]

Based on the positive results of a Phase II study by Azuma et al.,[24] a multicentre, double-blind, placebo-controlled, randomised Phase III clinical trial was conducted in Japan to determine the efficacy and safety of pirfenidone in 275 patients with IPF.[25] Patients were randomised to pirfenidone 1800 mg per day, pirfenidone 1200 mg per day or placebo using a 2:1:2 ratio, with 267 patients evaluated for the efficacy of pirfenidone. The dose of pirfenidone was increased in a stepwise manner up to the treatment dose over four weeks. The primary endpoint was vital capacity (VC) from baseline to 52 weeks. This was changed before unblinding of the study (it was previously the lowest arterial oxygen saturation measured by pulse oximetry (SpO_2_) during the six-minute steady state exercise test). This decision was based on the evolved knowledge of assessment with objective measurements in IPF, along with the lack of validation of the steady state exercise test and problems in reproducing SpO_2_ measurements. Secondary endpoints included progression-free survival (this was defined as time until the first progressive event, i.e. either decrease in VC of >10% or death) and change in the lowest SpO_2_ during the six-minute steady state exercise test.[25]

Statistically significant differences were observed between the pirfenidone 1,800 mg group and the placebo group for both the primary and secondary endpoints. Pirfenidone was associated with a 44% reduction in the VC decline compared with placebo (-0.09 L vs -0.16 L; p=0.0416), along with a significant increase in progression-free survival (p=0.0280).[25] Pirfenidone was relatively well tolerated, the most common adverse event observed with pirfenidone was photosensitivity, which was rated as mild in the majority of patients,[25] and has previously been documented as a side effect associated with pirfenidone treatment.[25,27] The data from this Phase III trial led to the approval of pirfenidone in Japan in 2008 for the treatment of IPF.

Two concurrent, similarly designed Phase III trials (studies 004 and 006, the “CAPACITY” studies), were conducted at 110 sites across North America, Australia and 11 European countries. Both were randomised, double-blind, placebo-controlled studies with treatment periods of 72 weeks.[26] The studies were designed to confirm the results of a Phase II study suggesting that pirfenidone reduced the deterioration in lung function in patients with IPF.[24]

Patients aged 40–80 years with mild-to-moderate IPF, diagnosed within the previous 48 months, were randomised to treatment with either oral pirfenidone or oral placebo. In study 004, patients were assigned to pirfenidone 2403 mg/day, 1197 mg/day or placebo in a 2:1:2 ratio. In study 006, patients were assigned to pirfenidone 2403 mg/day or placebo in a 1:1 ratio. Pirfenidone was administered with food three times a day and increased to the full dose (2403 mg/day) over two weeks. The lower dose of 1197 mg/day was included in study 004 to investigate any dose-response effect in terms of efficacy.[26]

The primary endpoint of both studies was change in percentage predicted FVC from baseline to week 72. Secondary endpoints at week 72 included categorical decline in FVC ≥10%, progression-free survival (time to confirmed ≥10% decline in percentage predicted FVC, ≥15% decline in percentage predicted DL_CO_ or death), mean change in 6MWT distance, mean change in percentage predicted DL_CO_, mean change in dyspnoea score, mean percentage change in worst SpO_2_ during 6MWT and time to worsening of IPF. Mortality was included as an exploratory endpoint. Categorical change in high-resolution computed tomographic (HRCT)-diagnosed fibrosis was included as a secondary endpoint in study 006.[26]

In study 004, pirfenidone 2403 mg/day significantly reduced mean decline from baseline to week 72 in percentage predicted FVC, compared with placebo (-8.0% [±16.5] vs -12.4% [±18.5], respectively; p=0.001), as well as the proportion of patients with FVC decline ≥10%. This treatment effect was evident between weeks 24 and 72. A pirfenidone effect was confirmed (p=0.0007) after repeat-measured analysis of the predicted percentage change in FVC across all assessment timepoints. In the pirfenidone 1197 mg/day group, the primary endpoint outcomes were intermediate to those of the 2403 mg/day pirfenidone and placebo groups.[26]

While the difference between groups in mean FVC change at Week 72 was not significant in Study 006 (-9.0% [SD 19.6] and -9.6% [19.1] respectively, p=0.501), this may have been due to a lower than expected rate of FVC decline in Study 006 after 1 year in the placebo group.[26] Moreover, a consistent pirfenidone effect was apparent until Week 48 (p=0.005) and also in an analysis of all study timepoints (p=0.007). Thus, the data from this study generally supported those from Study 004, with a positive treatment effect of pirfenidone being observed at all timepoints from weeks 12 to 48 but not at later time points.[26]

The effect of pirfenidone treatment on percentage predicted FVC at week 72 was supported by pooled analysis of data from both studies. Mean decline in percentage predicted FVC was -8.5% and -11.0% for the pirfenidone 2403 mg/day and placebo groups, respectively (p=0.005). Additionally, the pooled analysis demonstrated a 30% reduction in the percentage of patients with a categorical decline in FVC ≥10% at week 72 (p=0.003), a 31% reduction in the mean decline in 6MWT distance (p>0.001) and a 26% reduction in the risk of death or disease progression (HR 0.74; 95% CI 0.57, 0.96; p=0.025).[26]

Exploratory analysis of mortality data revealed that the hazard ratios for all-cause mortality (p=0.315) and mortality related to IPF at any time during the study (p=0.117), although not significant, numerically favoured pirfenidone over placebo. This was also the case with on-treatment IPF-related mortality, which occurred in 3% of patients treated with pirfenidone and 7% of those given placebo (p=0.03).[26]

## Cochrane meta-analysis of treatment effect

Meta-analyses performed by the Cochrane Collaboration, published in 2010, investigated the treatment effect of pirfenidone using data from the clinical trials performed to date. Data from the two Japanese studies were eligible for a meta-analysis as they both included the endpoint of absolute change in VC.[28] A statistically significant difference was observed in terms of decline in VC in favour of pirfenidone, underlining the beneficial effect of pirfenidone on the change in VC compared to baseline. As progression-free survival was also used as an endpoint in the Phase III study by Taniguchi et al,[25] it was possible to combine the data from this study and perform a meta-analysis with data from the CAPACITY studies. The overall result of this meta-analysis suggested that pirfenidone reduced the risk of disease progression by 30% (HR 0.70, 95% CI 0.56 to 0.88) in patients with IPF.[28]

## Tolerability in patients with IPF

Regarding safety, pirfenidone was shown to be generally well tolerated at the 2403 mg/day dose in the CAPACITY studies.[26] There was no significant difference in the number of patients experiencing serious treatment-emergent adverse events between the pirfenidone (pooled data) and placebo groups (33% and 31% respectively). The majority of patients treated with pirfenidone 2403 mg/day experienced at least one treatment-emergent adverse event, with the most common adverse events being gastrointestinal, skin disorders and dizziness. These adverse events were consistent with the known safety profile of pirfenidone and were usually mild to moderate in severity.[24] Adverse events leading to discontinuation occurred in 15% of pirfenidone-treated patients and 9% of placebo-treated patients. The most common cause of study discontinuation was IPF (3% of patients in each group). The only other causes of treatment discontinuation in the pooled pirfenidone group was nausea (1%) and rash (1%).[26]

An extension phase of the CAPACITY studies (called RECAP) was designed to assess the safety of pirfenidone beyond the duration of the Phase III studies. At Week 72 of the RECAP extension study, patients had been treated with pirfenidone for a mean duration of 2.9 years (range, 1-4). A number of patients (n=114) had been treated at the full dose for at least three years. Data from the RECAP extension study confirm the tolerability of pirfenidone.[29]

Common adverse events and those considered treatment-emergent occurred in a very similar proportion of patients to those reported during the CAPACITY studies. Almost all patients (98%) reported at least one treatment-emergent adverse event, compared with 99% of patients in the CAPACITY studies across both treatment arms. Similar proportions of patients in RECAP experienced serious adverse events to those in the CAPACITY studies (33% vs 33%). The incidence of common adverse events was very similar to that observed in the CAPACITY studies, and were generally mild to moderate in severity. No new or unexpected safety issues were observed.[29]

Rash or photosensitivity occurred in fewer patients from the RECAP extension study than in the CAPACITY studies (20% vs 44%). This was more common among patients initiating treatment with pirfenidone compared with those who were continuing with treatment (28% vs 12%). These data provide further important information on treatment with pirfenidone and demonstrate its tolerability.[29]

## Conclusions

There has been a considerable advance in terms of research into prognostic factors, with decline in % FVC being found to be a predictor of mortality risk. Until recently, therapeutic developments had lagged somewhat, but the increase in the number of clinical trials has been encouraging. However, many of these trials either failed to show significant treatment benefit against this challenging disease. Further studies are required to evaluate the potential benefit of other agents, such as N-acetylcysteine (NAC) [30] and nintedanib (BIBF 1120),[31] in IPF. The first major step forward has been the European approval of pirfenidone for patients with mild-to-moderate IPF. Pirfenidone has demonstrated statistically-significant and clinically-meaningful effects in clinical trials. Overall, pirfenidone provides a significant treatment benefit for patients with IPF and represents an appropriate option as first-line therapy for these patients.

## Abbrevations

## Competing interests

Vincent Cottin has received fees for speaking from Intermune, Boehringer Ingelheim, and Actelion, and has participated as a member of steering committees, a member of data safety monitoring boards or as an investigator to clinical trials sponsored by Actelion, Boehringer Ingelheim, Gilead, and Intermune Inc.

### Acknowledgements

The author thanks C. Trenam, I. Mandic and M. Smith of IntraMed Communications for editorial assistance in the preparation of the manuscript. Development of this article was supported by InterMune AG.

## Declarations

This article has been published as part of *Respiratory Research* Volume 14 Supplement 1, 2013:IPF in 2011 – Key updates on guidelines and therapeutics. The full contents of the supplement are available online at http://respiratory-research.com/supplements/14/S1. Publication of this supplement was supported by IntraMed Communications with funding from InterMune, AG. InterMune is the manufacturer of pirfenidone, a product mentioned in this article. The supplement originates from presentations given at the “AIR Event: Advancing IPF Research. Working together to translate IPF research into practice” held in Berlin in November 2011. The publication was proposed by IntraMed Communications and developed in consultation with the journal. All articles in the supplement have undergone the journal’s standard peer review process.
